# Organoid models of ovarian cancer: resolving immune mechanisms of metabolic reprogramming and drug resistance

**DOI:** 10.3389/fimmu.2025.1573686

**Published:** 2025-03-21

**Authors:** Lanyue Zhang, Jiangnan Zhao, Chunyu Su, Jianxi Wu, Lai Jiang, Hao Chi, Qin Wang

**Affiliations:** ^1^ Clinical Medical College, Southwest Medical University, Luzhou, China; ^2^ Department of Preventive Medicine, Southwest Medical University, Luzhou, China; ^3^ Sichuan Provincial Center for Gynecology and Breast Diseases (Gynecology), Affiliated Hospital of Southwest Medical University, Luzhou, China

**Keywords:** ovarian cancer, organoid, drug resistance, metabolic reprogramming, molecular mechanisms, immune escape, personalized therapy

## Abstract

Metabolic reprogramming is a hallmark of ovarian cancer, enabling tumor progression, immune evasion and drug resistance. The tumor microenvironment (TME) further shapes metabolic adaptations, enabling cancer cells to withstand hypoxia and nutrient deprivation. While organoid models provide a physiologically relevant platform for studying these processes, they still lack immune and vascular components, limiting their ability to fully recapitulate tumor metabolism and drug responses. In this study, we investigated the key metabolic mechanisms involved in ovarian cancer progression, focusing on glycolysis, lipid metabolism and amino acid metabolism. We integrated metabolomic analyses and drug sensitivity assays to explore metabolic-TME interactions using patient-derived, adult stem cell-derived and iPSC-derived organ tissues. Among these, we found that glycolysis, lipid metabolism and amino acid metabolism play a central role in tumor progression and chemotherapy resistance. We identified methylglyoxal (MGO)-mediated BRCA2 dysfunction as a driver of immune escape, a role for sphingolipid signaling in tumor proliferation and a role for kynurenine metabolism in CD8+ T cell suppression. In addition, PI3K/AKT/mTOR and Wnt/β-catenin pathways promote chemoresistance through metabolic adaptation. By elucidating the link between metabolic reprogramming and immune evasion, this study identifies key metabolic vulnerabilities and potential drug targets in ovarian cancer. Our findings support the development of metabolically targeted therapies and increase the utility of organoid-based precision medicine models.

## 1 Introduction

Ovarian cancer is a deadly gynecologic disease, causing over 152,000 deaths annually (1). While most patients initially respond to platinum-based therapies, resistance mechanisms, including paclitaxel resistance, hinder treatment progress (2, 3). This highlights the urgent need to study ovarian cancer metabolism and drug resistance to achieve precision therapy (3). However, ovarian cancer’s high heterogeneity complicates research (4). Organoid technology preserves the tumor microenvironment (TME), mimics tumor heterogeneity (5–7). It allows the study of developmental and pathogenic pathways that traditional animal models struggle to replicate and supports real-time imaging at lower costs and higher throughput than animal models, with a broader range of cell types than 2D cultures (8, 9). Additionally, it simulates drug penetration and metabolism, aiding in personalized therapy (10).

In ovarian cancer, dysregulated signaling pathways and metabolic reprogramming empower cancer cells to proliferate and persist within the adverse tumor microenvironment (TME) (11). Various signaling pathways control cell proliferation, survival, and stemness, while metabolic reprogramming closely links to cisplatin resistance (12–14). ovarian cancer organoids provide a realistic model to study these pathways and processes, enhancing the understanding of drug resistance mechanisms (15). This paper examines different drug-related metabolic and signaling pathways in ovarian cancer organoids, summarizes drug resistance, and discusses limitations in personalized treatment to inform clinical applications.

## 2 Different sources of ovarian cancer-like organs

Organoids are tissue analogs with a certain spatial structure formed by *in vitro* three-dimensional culture using adult stem cells, pluripotent stem cells, or patient-derived cells (16, 17).They have stable biological characteristics: tumor-derived organoids accurately replicate their structural, phenotypic, and genetic features, have similar tumor heterogeneity, in TME effectively mimic immune cells and stromal components, can be cultured *in vitro* long-term culture (17), and the ability to preserve metabolic adaptations in tumors, dynamically regulate glycolysis, lipid metabolism and amino acid metabolism, to study metabolic reprogramming in a way that more closely approximates the *in vivo* microenvironment, and to generalize the formation of two events occurring in organoid bodies, namely, the cellular adhesion of categorical aggregates and the spatially specific cellular hematopoietic stereotypes (18). (**Table 1**).

**Table 1:** Organoid modeling in ovarian cancer.

Organoid source	Genetic mutation	Specificities	Related Metabolic Mechanisms	References
iPSC source	BRCA1	iPSC-like organs mimic the transformation of fallopian tube epithelium to plasmacytoid ovarian cancer in patients with early-onset ovarian cancer and contain pluripotency markers (OCT4, SOX2, etc.)	Dependence on Wnt signaling to enhance stem cell properties	(19, 21)
ASC source	FBXW7	Heterogeneity and subclonal evolutionary capacity of the primary tumor is preserved and responds differently to platinum-based drugs	Can be used to assess chemotherapy resistance and capture resistance by different tumor subtypes	(22, 23, 115, 116)
Patient-derived PDO	TP53 and BRCA mutations	OC PDO preserves genomic characteristics of the original tumor and drug response is consistent with the patient	Preservation of TME features to mimic HGSC heterogeneity	(3)
High-grade plasmacytoid carcinoma (HGSOC)	TP53 and BRCA mutations	TP53 mutations, DNA repair defects and genomic instability	HGSOC contains genes from BRCA1 or BRCA2 mutant lines with specific types of DNA repair defects (HR defects)	(3, 28, 117)

### 2.1 Ovarian cancer-like organs derived from induced pluripotent stem cells

Induced pluripotent stem cells (iPSCs) derived from patients with early-onset ovarian cancer carrying BRCA1 gene mutations possess the capability to differentiate into fallopian tube epithelial (FTE)-like structures (19). Pathogenic BRCA1 mutations promote the transformation of normal FTECs into high-grade serous ovarian cancer cells (20). Additionally, these induced pluripotent stem cells (iPSCs) exhibit embryonic stem cell-like morphology and display pluripotent characteristics, including RNA expression of key pluripotency factors and the production of pluripotency marker proteins (21).

### 2.2 Ovarian cancer-like organs derived from adult stem cells

Ovarian cancer-like organs derived from ASCs retain the intratumoral heterogeneity and subclonal evolutionary capacity of the primary tumor (21). Different tumor subtypes of these organoids have differential responses to standard platinum-based chemotherapy, including subtypes that become chemoresistant in recurrent ovarian cancer, e.g. PDO can be used in drug screening assays to determine and capture the response of different tumor subtypes to platinum-based chemotherapy (22, 23).

### 2.3 Patient-derived ovarian cancer-like organs

Ovarian cancer patient-derived organoids (OC PDOs), established from ovarian cancer patient specimens, preserve the genomic features of the original tumor, including mutations, copy number variations, and other genomic alterations (24).OC PDOs also replicate the heterogeneity of the original tumor and exhibit drug responses that align with the clinical outcomes observed in patients (25).

The fallopian tube is notably recognized as the primary tissue of origin for ovarian cancer, particularly high-grade serous ovarian carcinoma (HGSOC), with fallopian tube epithelial cells (FTEC) considered the cells of origin for HGSOC. These cells are enriched with stem cells dependent on Wnt signaling and have the ability to form organoids stably and efficiently (26). Patient-derived HGSOC organoids are morphologically and molecularly consistent with their parent tumors, exhibiting features of nuclear pleomorphism, prominent nucleoli and dense chromatin formed by TP53 mutations (27).

HGSOC frequently harbors BRCA1 or BRCA2 mutations and homologous recombination (HR) defects, leading to synthetic lethality in HR-deficient cells and sensitivity to PARPi (e.g., olaparib) (28). However, most HGSOC-like organoids possess functional HR repair, making them resistant to PARPi (27).

**Figure 1:**
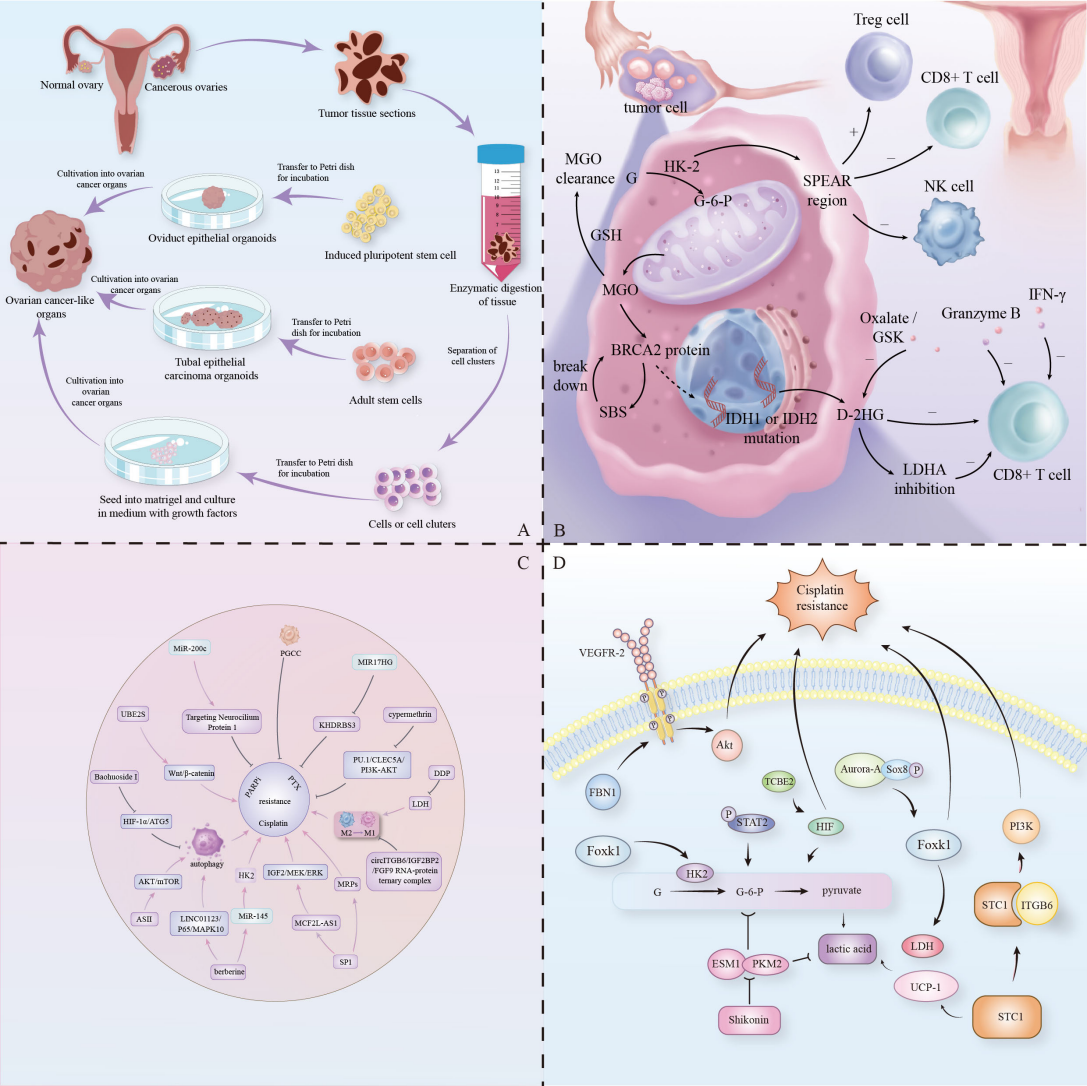
**(A)** Organoid models of ovarian cancer from three different sources. **(B)** Schematic diagram of the glycolytic metabolic pathway in ovarian cancer cells, with the dotted line indicating the normal pathway in the absence of SBS action. **(C)** Chemotherapeutic agents and their targets. Mechanisms of action of chemotherapeutic agents and networks of resistance-related gene and protein interactions. **(D)** Mechanisms of metabolic regulation by signaling pathways.

Both HGSOC and their organoids exhibit genomic instability, TP53 mutations, and distinct mRNA and miRNA expression profiles (29, 30). While many differentiated HGSOC-like organoids lack the patient’s immune microenvironment and vascular system, some newer versions show histological and molecular diversity, including CD34+ endothelial cells that retain critical immune and vascular structures (31). This enhancement better replicates tumor complexity, providing a more effective model for studying HGSOC pathomechanisms. (**Figure 1A**)

## 3 Mechanisms of basal metabolic regulation in ovarian cancer-like organs

Metabolic reprogramming, a hallmark of ovarian cancer, allows cells to adjust energy metabolism for rapid proliferation and survival in diverse conditions (32). Ovarian cancer cells typically achieve metabolic pattern remodeling by enhancing glycolytic activity and inducing the Warburg effect (33). Additionally, these cells can regulate fatty acid and amino acid metabolism and inhibit oxidative phosphorylation to meet their growth requirements.

### 3.1 Glycolysis

#### 3.1.1 MGO

Methylglyoxal (MGO) as a highly reactive dicarbonyl metabolite. It is an important endogenous dicarbonyl metabolic component in the human body and is present in many different tissues and organs (34).

As obtained in studies of non-malignant breast cells or patient-derived organ tissues, MGO induces cancer-associated mutant single base substitutions (SBS), triggers BRCA2 proteolysis, and transiently disables the tumor-suppressor function of BRCA2 in DNA repair and replication, leading to functional haploinsufficiency (35, 36). According to Knudson’s “two strikes” theory, tumor development usually requires two mutations: the first mutation may be inherited, and the second occurs in somatic cells (37). And defects in functional haploinsufficiency mean that even without complete loss of complete mutations in both genes, tumors may still result. This approach bypasses Knudson’s “double whammy” requirement for tumor initiation in the presence of partially preserved BRCA2 function (38).

Smith-Beckerman DM et al. reported that glyoxalase 1 (Glo1) is upregulated in FH-OSE cells of women with ovarian cancer carrying a BRCA1 mutation. Glo1 plays a key role in clearing methylglyoxal (MGO), catalyzing its conjugation with GSH to form less toxic products (39). This suggests that MGO accumulation in ovarian cancer may serve a protective role when BRCA2 is impaired, potentially impacting BRCA2 function and influencing ovarian cancer progression through Glo1 regulation. However, the role of Glo1 in MGO metabolism suggests that MGO may affect BRCA2 function, but whether it directly induces BRCA2 degradation requires further experimental verification.

#### 3.1.2 D-2HG

D-2HG is a metabolite produced primarily by isocitrate dehydrogenase (IDH) mutant tumor cells (40). In TME, D-2HG significantly weakened the defense function of nearby CD8+ T cells and acutely impaired their antitumor activity, including decreasing granzyme B expression and inhibiting IFN-γ release, which further weakened their immune response (41, 42). In addition, D-2HG inhibits the proliferation and cytokine (e.g., IL-2, IFN-γ, TNF-α) production of immune cells by inhibiting LDH activity, disrupting glycolytic fluxes and NAD+/NADH balance, thereby reducing their ability to kill cancer cells (43, 44). D-2HG also modifies the glucose metabolism of CD8+ T cells, further inhibiting their proliferation and cytotoxicity (42, 45, 46).

D-2HG accumulation is usually caused by LDH1 or LDH2 mutations. In EOC cells, low LDH1 expression leads to D-2HG accumulation, which inhibits CD8+ T cell proliferation and cytotoxicity and promotes immune escape (47). It has been shown that the use of LDH inhibitors (e.g., oxalate and GSK) significantly reduces D-2HG production in samples from glioma patients with LDH1 mutations, thereby inhibiting immune escape and delaying tumor progression. It was also demonstrated that LDH2 enhances metabolic remodeling and promotes OC growth by enhancing the activity of the TCA cycle (48). These results suggest that in ovarian cancer-like organs, LDH inhibitors may restore T cell activity and enhance tumor immune response by reducing D-2HG concentration (42).

#### 3.1.3 Hexokinase 2

Hexokinase-2 (HK2), which catalyzes the first step of glycolysis, is highly expressed in ovarian cancer cells (49). It was shown that HK2 supports rapid proliferation and survival of tumor cells by promoting glycolysis and enhancing cellular energy production (50). In the presence of sufficient oxygen, tumor cells increase glucose uptake and tend to ferment glucose into lactic acid, a phenomenon known as the “Warburg effect” (51). HK2 induces it, resulting in the formation of a more acidic environment around the cancer cells (50). This acidic microenvironment suppresses immune cell function through a combination of direct and indirect mechanisms. Lactate accumulates in the tumor microenvironment and binds to GAPDH, thereby directly inhibiting immune cell function and proliferation (52). In addition, the acidic environment not only inhibits the production of cytokines associated with anti-tumor immune responses, but also enhances the migration of tumor cells by decreasing the migration and infiltration ability of the immune cells while enhancing their migration (53–56). Together, these factors make it more difficult for immune cells to effectively reach and function at the tumor site. In addition, the acidic environment can promote the proliferation and activation of regulatory T cells (Tregs), thus indirectly inhibiting the function of CD8+ T cells and NK cells (57, 58). The acidic environment reduces the viability and killing ability of anti-tumor immune cells (e.g., CD8+ T cells and NK cells) and promotes the proliferation of regulatory T cells (Tregs) (59, 60). Therefore, it can be hypothesized that the HK2-induced Warburg effect can regulate the CD8+ and NK cells through acidifying the microenvironment in ovarian carcinoma carcinoids (51).

In ovarian cancer cells, there exists a negative regulatory relationship between AMPK phosphorylation and HIF-1α protein (61, 62). The activation of AMPK inhibits glycolysis and further suppresses the function of HIF-1α by promoting its degradation (63). However, the overexpression of HIF-1α can conversely inhibit the activation of AMPK and prevent the metabolic shift from glycolysis to OXPHOS (64). Therefore, the proliferation of ovarian cancer cells can be inhibited by silencing the activation of AMPK and suppressing the HIF-1α protein through transient receptor potential melastatin 7 (TRPM7), thereby adjusting the metabolic network and shifting the preferred glycolysis to oxidative phosphorylation (OXPHOS) (65, 66).

### 3.2 Lipid metabolism

Sphingolipids are mainly composed of sphingomyelin and fatty acids, which are widely present in cell membranes (67). synthesizing sphingolipids from scratch is an important pathway for tumor immune evasion. Sphingolipids, as specific sphingolipids, can promote cell growth and survival in cancer cells (68).

The hypoxic tumor microenvironment induces the expression of the secreted protein ESM1 through the transcription of HIF-1α (57, 69), which in turn promotes the SUMOylation of PKM2 and the subsequent formation of PKM2 dimers (70). This process promotes the Warburg effect and the nuclear translocation of PKM2, which ultimately leads to STAT3 phosphorylation (71, 72). Enhancement of fatty acid synthesis in ovarian cancer (70). Because it was found that shikonin inhibits the molecular interaction between ESM1 and PKM2, thus preventing the formation of PKM2 dimers and inhibiting glycolysis and fatty acid synthesis in ovarian cancer (70).

### 3.3 Amino acid metabolism

Amino acid metabolism regulates immune escape by modulating immune cell function (73, 74). For example, the Kynurenine pathway in tryptophan metabolism directly promotes immune escape by activating Treg cells and inhibiting CD8+ T cell activity (45, 46, 75). Arginine promotes T cell and NK cell function, resulting in enhanced proliferation, differentiation and effector function, thereby increasing the anti-tumor activity of immune cells (76, 77).

Therefore, it can be inferred that in the study of ovarian cancer-like organs, the survival of tumor cells can be effectively interfered with by regulating the amino acid metabolic pathway. For example, interfering with tyrosine catabolism in epithelial ovarian cancer (EOC) as the main disease model can inhibit DNA damage caused by genotoxic chemotherapeutic drugs (78). Depletion of fumarate acetoacetate hydrolase (FAH), a key enzyme in tyrosine metabolism, reduces the sensitivity of EOC to chemotherapy (78). In addition, tyrosine metabolism also plays an important role in tumor immune escape. Tyrosine can be converted to tryptophan, which in turn affects the activity of the Kynurenine pathway and promotes tumor cell immune escape (75, 79). Therefore, intervening in tyrosine metabolism may not only enhance the anti-tumor response of immune cells, but also improve the efficacy of chemotherapeutic agents and enhance the therapeutic effect. (**Figure 1B**)

## 4 signaling pathways in ovarian cancer-like organs

The tumor microenvironment (TME) consists of a wide range of non-cancer cells, non-cellular components and molecules released by them (80, 81), and is characterized by hypoxia, low pH and high redox state (82). However, cancer cells can survive in this hypoxic, nutrient-depleted environment (83). In particular, ovarian cancer cells often adapt to this microenvironment by enhancing glycolysis, and this metabolic reprogramming also enables tumor cells to rapidly produce energy and metabolic intermediates that support tumor cell proliferation and survival (11).

### 4.1 Glycolysis

Multiple signaling pathways regulate glycolysis, impacting tumor proliferation, migration, survival, and resistance to chemotherapy. For instance, serine/threonine kinase Aurora-A binds to the transcription factor SOX8, phosphorylating its Ser327 site, which increases FOXK1 expression and regulates genes linked to cellular senescence and glycolysis, such as LDHA and HK2. This promotes glucose metabolism and induces cisplatin resistance. Similarly (84), the TCEB2/HIF1A axis enhances cisplatin resistance by promoting glycolysis and angiogenesis (85). Furthermore, increased FBN1 expression in cisplatin-resistant ovarian cancer models indicates that the Fibrillin-1/VEGFR2/STAT2 axis plays a regulatory role in glycolysis and angiogenesis, thereby contributing to cisplatin resistance (86).

### 4.2 Fat metabolism

Fatty metabolism also undergoes metabolic reprogramming (11). For example, STC1 in the FOXC2/ITGB6 signaling axis can promote lipid metabolism by up-regulating lipid-related genes such as mitochondrial brown lipolytic coupling protein 1 (UCP1), TOM20 and perilipin1 (87). ESM1 promotes fatty acid synthesis and angiogenesis in ovarian cancer through the PKM2-dependent Warburg effect within a hypoxic TME, while shikonin effectively disrupts the interaction between ESM1 and PKM2, subsequently inhibiting glycolysis, fatty acid synthesis, and angiogenesis in ovarian cancer (70). (**Figure 1D**)

## 5 Modulating effects of chemotherapeutic agents

### 5.1 Basic metabolism

Berberine, an alkaloid with antitumor properties, inhibits the Warburg effect in ovarian cancer cells by enhancing TET3-mediated demethylation via the TET3/miR-145/HK2 signaling pathway (88). Moreover, berberine modulates autophagy and glycolysis in ovarian cancer through the LINC01123/P65/MAPK10 signaling axis (89).

Cryptotanshinone (CT) can inhibit cancer growth by inhibiting glycolysis and oxidative phosphorylation (90). Isovalerolactone, has anticancer activity. It has been shown to inhibit glycolysis and suppress cisplatin resistance in ovarian cancer cells (91). LDH can repolarize macrophages from a tumor-promoting M2 phenotype to a tumor-suppressive M1 phenotype by facilitating this phenotypic shift (92). Conversely, the platinum complex (DDP) downregulates the expression of LDHA and LDHB, thereby inhibiting glycolysis and glucose oxidation (93).

### 5.2 Signaling pathways

FOXO1 acts as a cell-specific coregulated transcription factor (94). For instance, within the miRNA-374a/FOXO1 signaling axis, propofol induces cell cycle arrest and decreases ovarian cancer cell viability by downregulating miR-374a, thereby alleviating its inhibition of FOXO1 (95). Additionally, FOXO1 influences ovarian cancer cell proliferation, migration, and invasion through apoptosis regulation in the EZH2/FOXO1 pathway (96). As an antitumor flavonoid, baicalein promotes apoptosis through the inhibition of the EZH2/FOXO1 signaling pathway (97).

The PI3K/AKT/mTOR pathway is a highly active cell signaling cascade in advanced ovarian cancer (12, 98). Xanthopterin suppresses ovarian cancer proliferation and cisplatin resistance by targeting the PU.1/CLEC5A/PI3K-AKT pathway (99). Ropivacaine, on the other hand, inhibits stemness and accelerates iron death of ovarian cancer cells by inactivating the PI3K/AKT signaling pathway (100).

### 5.3 Signal regulation in drug resistance mechanisms

The transcription factor SP1 enhances cisplatin resistance through multiple mechanisms. Beyond SP1-induced MCF2L-AS1, which activates the IGF2/MEK/ERK pathway (101), SP1 also transcribes multidrug resistance-associated proteins (MRPs) and key enzymes involved in arachidonic acid (AA) metabolism (e.g., 12-lipoxygenase), thereby promoting chemoresistance and metastasis in ovarian cancer (102). Moreover, circITGB6 promotes cisplatin resistance in ovarian cancer by forming the circITGB6/IGF2BP2/FGF9 RNA-protein ternary complex, which facilitates the polarization of tumor-associated macrophages (TAMs) towards the M2 phenotype (103, 104).

We also identified ex vivo and *in vivo* factors such as miR-219-5p, Baohuoside I, and Astragaloside II (ASII) that could modulate cisplatin resistance by regulating autophagy in ovarian cancer cells. ASII can induce autophagy by inhibiting the AKT/mTOR signaling pathway (105), while Boswellia serrata I suppresses autophagy through downregulation of the HIF-1α/ATG5 signaling axis (106). Moreover, miR-219-5p inhibits Wnt/β-catenin signaling and autophagy in ovarian cancer cells by targeting HMGA2 (105) and reduces cisplatin resistance by suppressing Wnt/β-catenin signaling and autophagy through HMGA2 targeting in these cells (107).

PARPi resistance involves various heterogeneous mechanisms, making it crucial to understand its molecular basis in patients to address chemoresistance and refine personalized treatments (108). Utilizing organoid technology, researchers are creating patient-derived organoids to predict responses to PARP inhibitors (PARPi) and explore strategies to overcome drug resistance in ovarian cancer (109). Notably, the Wnt/β-catenin pathway contributes to resistance in both cisplatin and PARPi (107). For instance, the ubiquitin-conjugating enzyme E2S (UBE2S) promotes PARPi resistance in ovarian cancer by activating Wnt/β-catenin signaling (110). Additionally, targeting polyploid giant cancer cells (PGCC) in ovarian cancer organoids could enhance therapeutic efficacy and reduce PARPi resistance (111). (**Figure 1C**)

## 6 Conclusion and prospect

This review systematically explores the application of ovarian cancer organoid models in the study of metabolic reprogramming, immune escape and drug resistance mechanisms, introducing and integrating organoids into the metabolism-immunity-drug resistance interaction framework. Metabolic reprogramming serves as a crucial indicator of tumor progression and therapeutic resistance (112). Organoid-based drug screening is more accurate and physiologically relevant than traditional techniques and is more likely to identify new strategies for treating metabolic disorders in ovarian cancer.

There are still limitations to this approach. Current cancer organoid culture techniques lack control and reproducibility, requiring the development of stable, reproducible platforms (9). Additionally, organoid models often lack vascular networks and immune systems, failing to fully replicate *in vivo* organ structure and function (113). Organoid techniques are also more time-intensive than 2D cultures and often require specialized media and growth factors (114). Despite advances, organoids lack vascular and immune components, limiting their ability to mimic tumor microenvironments and drug responses, necessitating further improvements. In the future, we can integrate immune and matrix co-culture, microfluidic dynamic culture system, single cell metabolomics and clinical metabolism data to optimize model stability and physiological relevance, so that it can better simulate the *in vivo* metabolic environment and help precision medicine research.
